# α, ω-Cholesterol-Functionalized Low Molecular Weight Polyethylene Glycol as a Novel Modifier of Cationic Liposomes for Gene Delivery

**DOI:** 10.3390/ijms151120339

**Published:** 2014-11-06

**Authors:** Cui-Cui Ma, Zhi-Yao He, Shan Xia, Ke Ren, Li-Wei Hui, Han-Xiao Qin, Ming-Hai Tang, Jun Zeng, Xiang-Rong Song

**Affiliations:** 1 Department of Anesthesiology, State Key Laboratory of Biotherapy, West China Hospital, Sichuan University, Chengdu 610041, Sichuan, China; E-Mails: ccma2012@126.com (C.-C.M.); heyaode@163.com (Z.-Y.H.); xiashanshan@sina.com (S.X.); huiliwei.love@163.com (L.-W.H.); qin_hanxiao@163.com (H.-X.Q.); tangminghai1982@126.com (M.-H.T.); 2 Central Laboratory, Science Education Department, Chengdu Normal University, Chengdu 610041, Sichuan, China; 3 Department of Pharmaceutical Sciences, University of Nebraska Medical Center, Omaha, NE 68198, USA; E-Mail: renkemallee@gmail.com

**Keywords:** cholesterol, polyethylene glycol, cationic liposomes, gene delivery, folate ligand

## Abstract

Here, three novel cholesterol (Ch)/low molecular weight polyethylene glycol (PEG) conjugates, termed α, ω-cholesterol-functionalized PEG (Ch_2_-PEG_n_), were successfully synthesized using three kinds of PEG with different average molecular weight (PEG_600_, PEG_1000_ and PEG_2000_). The purpose of the study was to investigate the potential application of novel cationic liposomes (Ch_2_-PEG_n_-CLs) containing Ch_2_-PEG_n_ in gene delivery. The introduction of Ch_2_-PEG_n_ affected both the particle size and zeta potential of cationic liposomes. Ch_2_-PEG_2000_ effectively compressed liposomal particles and Ch_2_-PEG_2000_-CLs were of the smallest size. Ch_2_-PEG_1000_ and Ch_2_-PEG_2000_ significantly decreased zeta potentials of Ch_2_-PEG_n_-CLs, while Ch_2_-PEG_600_ did not alter the zeta potential due to the short PEG chain. Moreover, the *in vitro* gene transfection efficiencies mediated by different Ch_2_-PEG_n_-CLs also differed, in which Ch_2_-PEG_600_-CLs achieved the strongest GFP expression than Ch_2_-PEG_1000_-CLs and Ch_2_-PEG_2000_-CLs in SKOV-3 cells. The gene delivery efficacy of Ch_2_-PEG_n_-CLs was further examined by addition of a targeting moiety (folate ligand) in both folate-receptor (FR) overexpressing SKOV-3 cells and A549 cells with low expression of FR. For Ch_2_-PEG_1000_-CLs and Ch_2_-PEG_2000_-CLs, higher molar ratios of folate ligand resulted in enhanced transfection efficacies, but Ch_2_-PEG_600_-CLs had no similar in contrast. Additionally, MTT assay proved the reduced cytotoxicities of cationic liposomes after modification by Ch_2_-PEG_n_. These findings provide important insights into the effects of Ch_2_-PEG_n_ on cationic liposomes for delivering genes, which would be beneficial for the development of Ch_2_-PEG_n_-CLs-based gene delivery system.

## 1. Introduction

Cholesterol (Ch), an essential membrane component in higher eukaryotes, modulates functions of membrane proteins and participates in several membrane trafficking and transmembrane signaling processes [1]. Ch facilitates the formation of semi-permeable barriers between cellular compartments and regulates membrane fluidity [1,2]. Ch has been widely used for liposome preparation and other lipid-based drug delivery systems [3,4]. Furthermore, the hydroxyl group in Ch can be modified with other moieties [5,6]. Poly (ethylene glycol) (PEG) cholesterol conjugates (PEG-Ch) had been developed to enhance the stability and activity of liposomes and other lipid-based drug delivery systems [7,8,9]. PEG-Ch conjugates were further modified with targeting ligands or antibodies to increase the targeting efficacy of the delivery systems [10,11,12].

As a PEG-Ch conjugate, it has been reported that the hydrophobic groups in α, ω-Ch-modified PEG (Ch_2_-PEG_n_) were capable of inserting into the hydrophobic interior of lipid bilayers or membranes [13,14,15]. High molecular weight Ch_2_-PEG_n_ (PEG_30000_–PEG_35000_) had been used to prepare liposome gels [16,17] and core-shell emulsion particles [18]. However, there is no report about applying Ch_2_-PEG_n_ to gene delivery up to now.

For PEG conjugates, the molecular weight of PEG had a significant effect on the properties of the drug delivery systems containing them [7,19,20,21]. PEG_2000_ modified polyethylenimine (PEI) was more efficient than PEG_5000_ modified PEI as evaluated by *in vitro* gene transfer [22]. PEG_400_ significantly enhanced fractional laser-assisted drug delivery when compared with PEG_2050_ and PEG_3350_ [23].

Therefore, the purpose of the study was to explore the potential application of novel cationic liposomes (Ch_2_-PEG_n_-CLs) containing Ch_2_-PEG_n_ using three kinds of PEG with different average molecular weight (PEG_600_, PEG_1000_ and PEG_2000_) for gene delivery. The impacts of variation in PEG molecular weight on the properties, toxicities and gene delivery efficacies were determined. In addition, the gene delivery efficacy of Ch_2_-PEG_n_-CLs was further investigated by introduction of the targeting moiety (folate ligand) in both folate-receptor (FR) overexpressing SKOV-3 cells and A549 cells with low expression of FR.

## 2. Results and Discussion

### 2.1. Synthesis and Identification of Ch_2_-PEG_n_

**Scheme 1:**
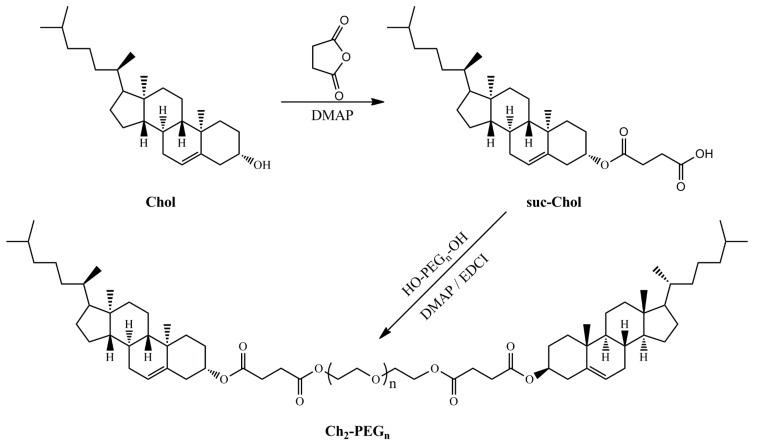
Synthesis route of Ch_2_-PEG_n_ conjugates.

**Figure 1:**
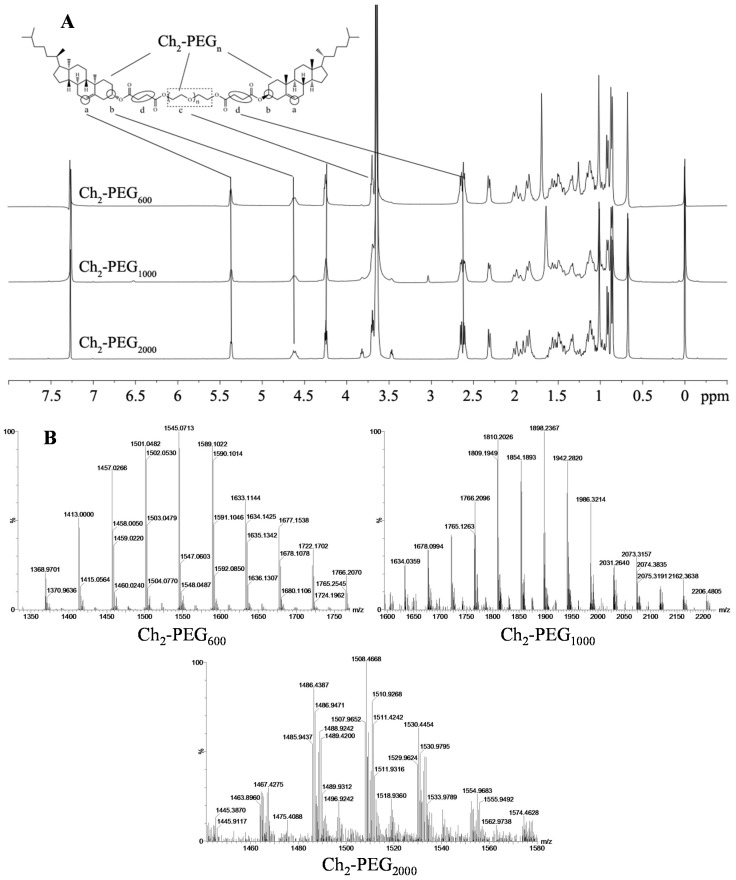
^1^H-NMR and mass spectra of Ch_2_-PEG_n_. (**A**) ^1^H-NMR spectra (400 MHz) of Ch_2_-PEG_n_ in CDCl_3_; (**a**,**b**) 6- and 3-position protons in Chol; (**c**) protons of methylene in PEG and (**d**) methylene proton of succinyl group. The principal proton peaks of Chol-suc and PEG were found in Ch_2_-PEG_n_; (**B**) Mass spectra of Ch_2_-PEG_n_. The *m*/*z* ions of Ch_2_-PEG_600_ and Ch_2_-PEG_1000_ are singly charged molecular-related ions; the *m*/*z* ions of Ch_2_-PEG_2000_ are doubly charged molecular-related ions. The measured molecular weight of Ch_2_-PEG_600_, Ch_2_-PEG_1000_ and Ch_2_-PEG_2000_ were 1544, 1895 and 3012 Da, respectively.

The successful synthesis of Ch_2_-PEG_n_ was confirmed by ^1^H-NMR and mass spectra. As shown in Scheme 1, Ch_2_-PEG_n_ was synthesized by esterification of succinic anhydride-cholesterol (suc-Ch) with PEG using 4-dimethylaminopyridine (DMAP) and 1-ethyl-3-(3-dimethyllaminopropyl) carbodiimide hydrochloride (EDCI) as catalysts. The ^1^H-NMR spectra of Ch_2_-PEG_n_ were shown in Figure 1A. The principal peaks of suc-Ch and poly (ethylene glycol) (PEG) moieties were observed [8,24]. The molecular weights of Ch_2_-PEG_n_ were measured by the Quadrupole-Time of Flight (Q-TOF) mass spectra as shown in Figure 1B. For Ch_2_-PEG_600_ and Ch_2_-PEG_1000_, the mass-to-charge ratio (*m*/*z*) spectrums showed dominant ions at *m*/*z* 1545 and 1896. Their *m*/*z* ions were singly charged [(M + H)^+^]. Therefore, the measured molecular weight of Ch_2_-PEG_600_ and Ch_2_-PEG_1000_ were 1544 and 1895 Da respectively after subtracting H^+^. For Ch_2_-PEG_2000_, the *m*/*z* values differed by 0.5 Da as z, so the number of charges was equal to 2. The *m*/*z* ions were doubly charged [(M + 2H)^2+^]. The measured molecular weight of Ch_2_-PEG_2000_ was 3012 Da [(1508 − 2) × 2]. All the calculated molecular weights were consistent with the true molecular weights.

The melting points and appearances of Ch_2_-PEG_n_ were summarized in Table 1. Three kinds of Ch_2_-PEG_n_ had different melting points, which might impact the film-forming property of the mixed lipids and therefore influence the stability of cationic liposomes.

**Table 1:** The melting points and appearances of Ch_2_-PEG_n_. The melting point was recorded as the midpoint value in the melting temperature range to facilitate comparison.

Samples	Melting Point (°C)	Appearance (25 °C)
PEG_600_ → Ch_2_-PEG_600_	20 → 33 (↑)	Clear liquid → Clear semi-solid
PEG_1000_ → Ch_2_-PEG_1000_	33 → 40 (↑)	White paste → Clear solid
PEG_2000_ → Ch_2_-PEG_2000_	52 → 47.5 (↓)	White flake → White powder

### 2.2. Physicochemical Properties of Ch_2_-PEG_n_-CLs

**Figure 2:**
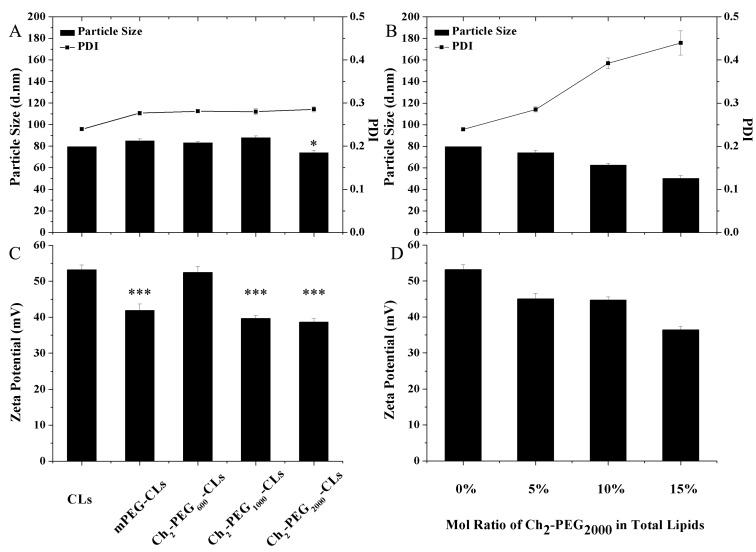
Particle size, PDI and zeta potential of Ch_2_-PEG_n_-CLs (Mean ± SD, *n* = 3, * *p* < 0.05, *** *p* < 0.001). (**A**,**C**) Particle size, PDI, and zeta potential of Ch_2_-PEG_n_-CLs, CLs (normal cationic liposomes without PEG introduction) and mPEG-CLs; (**B**,**D**) The effect of molar ratio of Ch_2_-PEG_2000_ on the particle size, PDI and zeta potential of Ch_2_-PEG_2000_-CLs.

The particle size and polydispersity index (PDI) of Ch_2_-PEG_n_-CLs were shown in Figure 2A. There were no significant differences of the particle size and PDI when comparing Ch_2_-PEG_600_-CLs and Ch_2_-PEG_1000_-CLs with mPEG-CLs (CLs modified by mPEG_2000_-suc-Ch). However, the particle size of Ch_2_-PEG_2000_-CLs (74 nm) was significantly smaller than those of Ch_2_-PEG_600_-CLs and Ch_2_-PEG_1000_-CLs (*p* < 0.05). It was considered that the size decrease of Ch_2_-PEG_2000_-CLs might be attributed to the introduction of Ch_2_-PEG_2000_, which anchored into the liposomal bilayer by Ch segments [25,26] and extended the PEG chain on the surface of the liposome. The PEG chain compressed the liposomal particle [22], and therefore reduced the particle size. Then the effect of Ch_2_-PEG_2000_ on particle size was further studied. As shown in Figure 2B, the particle size of Ch_2_-PEG_2000_-CLs gradually decreased with a higher polydispersity when the molar ratio of Ch_2_-PEG_2000_ increased. Due to the high curvature and micellar preference of the large hydrophilic PEG chain, it was challenging to stably integrate high molar ratio Ch_2_-PEG_n_ into liposomal bilayer [27]. Therefore, 5 mol% Ch_2_-PEG_2000_ was the optimal molar ratio.

The zeta potentials of Ch_2_-PEG_1000_-CLs and Ch_2_-PEG_2000_-CLs were significantly lower than that of Ch_2_-PEG_600_-CLs as seen in Figure 2C (*p* < 0.001). It suggested that Ch_2_-PEG_1000_ and Ch_2_-PEG_2000_ could shield the electric charge of cationic liposomes. Therefore, they were good candidates like mPEG_2000_-suc-Ch for preparing long-circulating or stealth liposomes. The zeta potential of Ch_2_-PEG_600_-CLs was close to CLs (Figure 2C), which was consistent with the previous report that low molecular weight PEG did not effectively shield the positive charge of cationic particles [19]. The potential impact of molar ratio of Ch_2_-PEG_2000_ on zeta potentials was also studied. As shown in Figure 2D, 15 mol % was the most efficient ratio in reducing zeta potential. Similar zeta potential values were found with 5 and 10 mol % Ch_2_-PEG_2000_.

### 2.3. Gene Transfection Efficiencies of Ch_2_-PEG_n_-CLs

**Figure 3:**
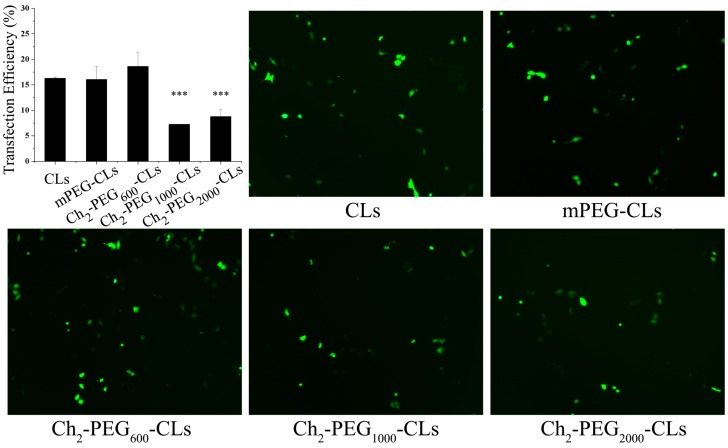
Transfection efficiency and fluorescence images (200×) of Ch_2_-PEG_n_-CLs (mean ± SD, *n* = 3, *** *p* < 0.001).

Different types of cationic liposomes were evaluated in gene transfection efficiencies by comparison. As shown in Figure 3, Ch_2_-PEG_600_-CLs had slightly higher transfection efficiency than CLs and mPEG-CLs, but there were no significant differences. In contrast, the transfection efficiencies of Ch_2_-PEG_1000_-CLs and Ch_2_-PEG_2000_-CLs were significantly lower than that of Ch_2_-PEG_600_-CLs (*p* < 0.001). The higher zeta potential of Ch_2_-PEG_600_-CLs (Figure 2C) than the other Ch_2_-PEG_n_-CLs might be due to its high transfection efficiency. Therefore, zeta potential is an important parameter when applying Ch_2_-PEG_n_ to gene delivery. Ch_2_-PEG_2000_-CLs, with the same zeta potential but the smaller particle size (Figure 2A), achieved a bit higher transfection efficiency than Ch_2_-PEG_1000_-CLs.

**Figure 4:**
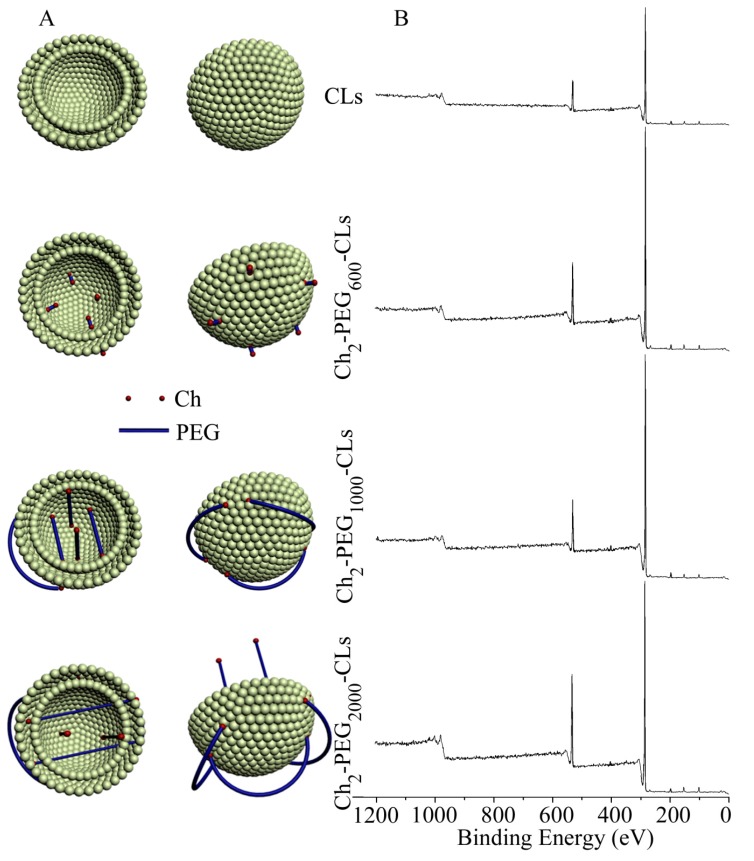
Schematic diagram and XPS analysis of Ch_2_-PEG_n_-CLs. (**A**) Ch segments of Ch_2_-PEG_n_ anchored into the lipid bilayer of CLs. Due to the short PEG chain, Ch_2_-PEG_600_ did not effectively shield the charge of CLs. Ch_2_-PEG_1000_ and Ch_2_-PEG_2000_ decreased the positive charge by covering the surface of CLs. With longer PEG chains, Ch_2_-PEG_2000_ compressed the liposomal particle and therefore Ch_2_-PEG_2000_-CLs showed a smaller particle size; (**B**) XPS analysis demonstrated that more PEG segments were located on the surface of liposomes when PEG molecular weight increased.

To further explain the differences in zeta potential and transfection efficacies of several cationic liposomes consisting of Ch_2_-PEG_n_ with different molecular weights, XPS analysis was carried out of various Ch_2_-PEG_n_-CLs and the schematic diagrams are shown in Figure 4. More oxygen atoms from ethylenedioxy groups of PEG were found on the surface of CLs with the increase of PEG molecular weight as shown in Figure 4B. The presence of PEG chains outside of CLs had the potential to shield the electronic charge, and decrease the zeta potential as shown in Figure 4A. However, Ch_2_-PEG_600_-CLs, with the shorter PEG chain, which could not shield the surface charge of liposomes, had a significant higher zeta potential than Ch_2_-PEG_1000_-CLs and Ch_2_-PEG_2000_-CLs, which resulted in the increased transfection efficiency. The zeta potentials of Ch_2_-PEG_1000_-CLs and Ch_2_-PEG_2000_-CLs were comparable and might be due to the saturation of the shielding effect. Ch_2_-PEG_1000_-CLs and Ch_2_-PEG_2000_-CLs also showed comparable transfection efficiency due to similar zeta potential.

### 2.4. Gene Transfection Efficiencies of F-Ch_2_-PEG_n_-CLs with Introduction of a Folate Ligand

The gene delivery efficacy of Ch_2_-PEG_n_-CLs was further examined by addition of a targeting moiety (folate ligand) in both folate-receptor (FR) overexpressing SKOV-3 cells and A549 cells with low expression of FR.

**Figure 5:**
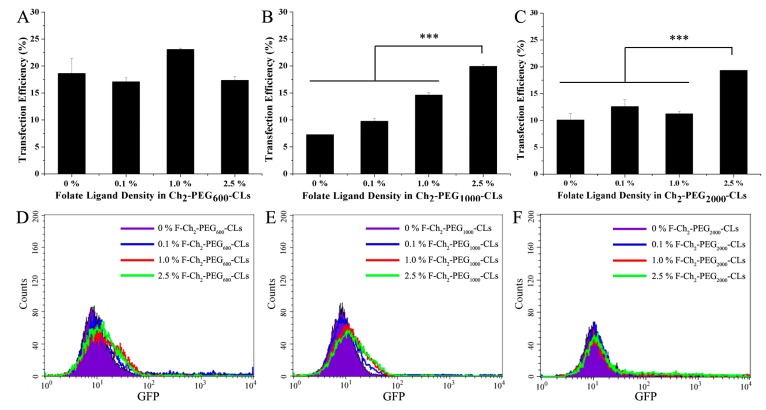
The effect of folate ligand densities in F-Ch_2_-PEG_n_-CLs on transfection efficiencies (mean ± SD, *n* = 3, *** *p* < 0.001). (**A**–**C**) The transfection efficiencies of F-Ch_2_-PEG_600_-CLs, F-Ch_2_-PEG_1000_-CLs and F-Ch_2_-PEG_2000_-CLs. 0%: Ch_2_-PEG_n_-CLs without folate ligand modification; 0.1%, 1.0% and 2.5%: Ch_2_-PEG_n_-CLs modified by 0.1, 1.0 and 2.5 mol % F-suc-PEG_2000_-Chol in total lipids, respectively; (**D**–**F**) The flow cytometry results of transfection by F-Ch_2_-PEG_n_-CLs.

We previously demonstrated that SKOV-3 cells overexpress the folate receptor alpha [24]. The effects of folate ligand and its density on the transfection efficiencies of Ch_2_-PEG_n_-CLs in SKOV-3 cells are shown in Figure 5. For Ch_2_-PEG_600_-CLs, the transfection efficacy was close to 19% without folate ligand modification. There was no special trend with the change of folate ligand ratio for F-Ch_2_-PEG_600_-CLs. Their transfection efficacies were all around 20% (Figure 5A,D). In contrast, transfection efficiencies of F-Ch_2_-PEG_1000_-CLs were significantly elevated with the increase of folate ligand density (*p* < 0.001, shown in Figure 5B,E). About 20% cells were transfected with 2.5% F-Ch_2_-PEG_1000_-CLs, which was almost 3-fold of that for Ch_2_-PEG_1000_-CLs without folate modification. Similarly, 2.5% F-Ch_2_-PEG_2000_-CLs had higher transfection efficiency than other Ch_2_-PEG_1000_-CLs as shown in Figure 5C,F. Therefore, the optimal ligand density should be screened when various PEG with different molecular weight was employed for preparing CLs in the future.

**Figure 6:**
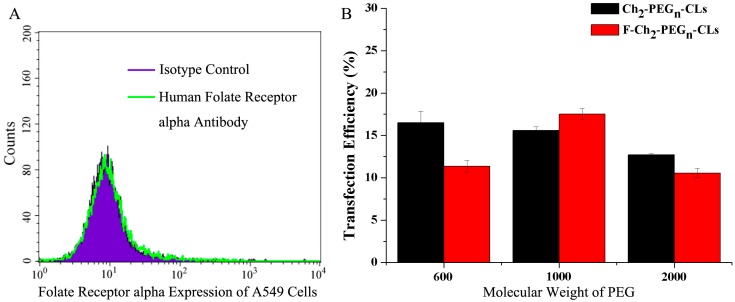
Folate receptor alpha expression and transfection efficiency on A549 cells. (**A**) Folate receptor alpha expression on A549 cells by flow cytometry and (**B**) The transfection efficiencies of Ch_2_-PEG_n_-CLs and F-Ch_2_-PEG_n_-CLs on A549 cells.

As shown in Figure 6A, folate receptor alpha is not expressed on A549 cells by folate receptor assay as previously reported [24]. When Ch_2_-PEG_n_-CLs and F-Ch_2_-PEG_n_-CLs were used to transfect A549 cells, F-Ch_2_-PEG_n_-CLs did not significantly increase the transfection efficiency of Ch_2_-PEG_n_-CLs as shown in Figure 6B.

### 2.5 Cytotoxicity Evaluation

**Figure 7:**
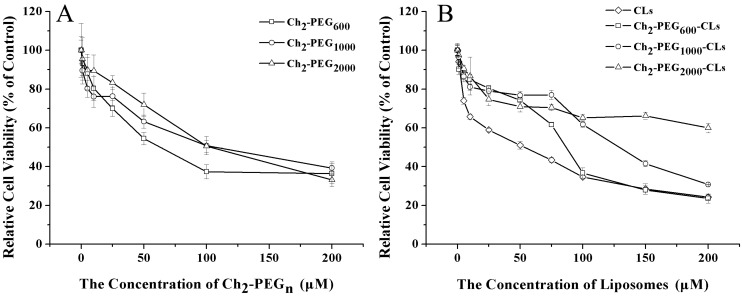
Cytotoxicity of Ch_2_-PEG_n_ and Ch_2_-PEG_n_-CLs on SKOV-3 cells by the MTT assay (Mean ± SD, *n* = 4–6). (**A**) IC_50_ values for Ch_2_-PEG_600_, Ch_2_-PEG_1000_ and Ch_2_-PEG_2000_ were about 78, 145 and 127 μM, respectively; (**B**) IC_50_ values for CLs, Ch_2_-PEG_600_-CLs, Ch_2_-PEG_1000_-CLs and Ch_2_-PEG_2000_-CLs were about 38, 100, 143 and 302 μM, respectively. The cytotoxicity of CLs was reduced by introducing Ch_2_-PEG_n_ into Ch_2_-PEG_n_-CLs.

The cytotoxicities of Ch_2_-PEG_n_ on SKOV-3 cells were shown in Figure 7A. The toxicities were concentration-dependent. After treatment for 24 h, the half maximal inhibitory concentration (IC_50_) for Ch_2_-PEG_600_, Ch_2_-PEG_1000_ and Ch_2_-PEG_2000_ on SKOV-3 cells were about 78, 145 and 127 μM, respectively. Ch_2_-PEG_600_ was slightly more toxic to SKOV-3 cells than the other. However, even for Ch_2_-PEG_600_, the IC_50_ value was higher than other PEG-lipid conjugates that have been extensively used for drug and gene delivery [24,28]. Therefore, Ch_2_-PEG_n_ may be one kind of safe material for gene delivery.

The cytotoxicities of Ch_2_-PEG_n_-CLs on SKOV-3 cells were shown in Figure 7B. The cytotoxicities gradually decreased with the increase of PEG molecular weight. The IC_50_ values for CLs, Ch_2_-PEG_600_-CLs, Ch_2_-PEG_1000_-CLs and Ch_2_-PEG_2000_-CLs were about 38, 100, 143 and 302 μM, respectively. 5 mol % Ch_2_-PEG_n_ significantly enhanced the safety of cationic liposomes when compared with CLs (*p* < 0.001). Therefore, employing Ch_2_-PEG_n_ in preparation was an effective way to reduce the cytotoxicity of cationic liposomes.

## 3. Experimental Section

### 3.1. Materials

Cholesterol (Ch) was obtained from Bio Life Science & Technology Co., Ltd. (Shanghai, China). Poly (ethylene glycol) (PEG) [molecular weight 600 (PEG_600_), molecular weight 1000 (PEG_1000_) and molecular weight 2000 (PEG_2000_)] and 3-(4, 5-Dimethyl-2-thiazolyl)-2, 5-diphenyl-2H-tetrazolium bromide (MTT) were purchased from Sigma-Aldrich (St. Louis, MO, USA). 1-(3-dimethylaminopropyl)-3-ethylcarbodiimide hydrochloride (EDCI) was provided by Accela ChemBio Co., Ltd. (Shanghai, China). 4-dimethylaminopyridine (DMAP) was obtained from AstaTech Pharma. Co., Ltd. (Chengdu, Sichuan, China). 1, 2-dioleoyl-3-trimethylammonium-propane (chloride salt) (DOTAP) was purchased from Avanti Polar Lipids Inc. (Alabaster, AL, USA). Folate-PEG-succinyl-cholesterol conjugate (F-PEG_2000_-suc-Ch) and mPEG-succinyl-cholesterol conjugate (mPEG_2000_-suc-Ch) were synthesized and purified in the same procedures as those recorded in our previous publications [8,24]. Green fluorescent protein plasmid DNA (pDNA) was extracted according to the EndoFree Plasmid Purification Handbook (QIAGEN, Hilden, Germany). All the other reagents and solvents were of analytical grade and were used without further purification except for chloroform used for cationic liposomes preparation.

### 3.2. Synthesis and Identification of Ch_2_-PEG_n_

#### 3.2.1. Synthesis of Ch_2_-PEG_n_

α, ω-Ch-modified PEGs (Ch_2_-PEG_n_) were synthesized according to Scheme 1. Firstly, Ch succinic anhydride ester (suc-Ch) was synthesized as described before [8]. In brief, Ch, succinic anhydride and DMAP were dissolved in dichlormethane and stirred for 48 h at room temperature. After removing the solvent, the crude product was washed by acetic acid. White suc-Ch was obtained. Secondly, PEG (PEG_600_, PEG_1000_, or PEG_2000_), suc-Ch, DMAP and EDCI were dissolved in chloroform. The mixture was refluxed for 72 h, concentrated under vacuum, and purified on a silica-gel column eluting with dichlormethane and methanol. Ch_2_-PEG_600_, Ch_2_-PEG_1000_ and Ch_2_-PEG_2000_ were obtained.

#### 3.2.2. ^1^H-NMR and Mass Spectra of Ch_2_-PEG_n_

^1^H-NMR spectra of Ch_2_-PEG_n_ were recorded on a Bruker ADVANCE^III^ spectrometer (400MHz) (Billerica, MA, USA) at room temperature. Ch_2_-PEG_600_, Ch_2_-PEG_1000_ and Ch_2_-PEG_2000_ were dissolved in CDCl_3_ with tetramethylsilane as the internal standard. The mass spectra of Ch_2_-PEG_n_ were measured using a Waters Q-TOF Premier (Milford, MA, USA) equipped with ion spray source and N_2_ as nebulization gas.

#### 3.2.3. Melting Point and Appearances

Melting points of Ch_2_-PEG_n_, PEG_1000_, PEG_2000_, mPEG_2000_-suc-Ch, suc-Ch and Ch were determined using SGW X-4 melting point apparatus (Shanghai Precision & Scientific Instrument CO., LTD., Shanghai, China). The appearances of the materials were recorded.

### 3.3. Preparation and Characterization of Cationic Liposomes

#### 3.3.1. Preparation of Liposomes

Ch_2_-PEG_n_-CLs were prepared by film dispersion method as described before [29]. In brief, DOTAP, Ch and Ch_2_-PEG_n_ (Ch_2_-PEG_600_, Ch_2_-PEG_1000_ or Ch_2_-PEG_2000_) at different molar ratios were dissolved in chloroform. Then the organic solvent was removed from the lipids solution using a Büchi rotary evaporator. A thin film was formed and further dried under high vacuum for 6 h at room temperature. The lipid film was hydrated with 5% (*w*/*v*) glucose solution and sonicated by a VCX130 Vibra-Cell (Sonics & Materials Inc., Newtown, CT, USA) until a translucent lipid suspension was obtained. Ch_2_-PEG_n_-CLs were formed. They were passed through a 0.22 μm Millipore microporous membrane and stored at 4 °C until use.

CLs and mPEG-CLs (served as controls), folate modified Ch_2_-PEG_n_-CLs (F-Ch_2_-PEG_n_-CLs, active targeted CLs) were prepared in the same way. CLs were made of DOTAP and Ch. mPEG-CLs were composed of DOTAP, Ch and mPEG_2000_-suc-Ch. F-Ch_2_-PEG_n_-CLs were consisted of F-PEG_2000_-suc-Ch, DOTAP, Ch and Ch_2_-PEG_n_.

#### 3.3.2. Size and Zeta Potential Determination

The mean particle size and zeta potential of the liposomes were measured by a Zetasizer Nano ZS ZEN 3600 (Malvern Instruments, Ltd., Malvern, Worcestershire, UK). The mean particle size was determined at a fixed angle of 173°. The zeta potential of 5 mg/mL liposome at pH 6.0 was automatically calculated from the electrophoretic mobility at 25 °C. All the experiments were performed in triplicate.

### 3.4. Cell Culture

Human ovarian carcinoma SKOV-3 cell line and human lung carcinoma A549 cell line were obtained from American Type Culture Collection (ATCC). Cells were cultured as a monolayer in Dulbecco’s Modified Eagles’s Medium (DMEM, Gibco, Carlsbad, CA, USA) or Roswell Park Memorial Institute medium (RPMI)-1640 medium supplemented with 10% fetal bovine serum, l-glutamine (2 mmol/L), penicillin (100 units/mL) and streptomycin (100 μg/mL) in a humidified atmosphere containing 5% CO_2_ at 37 °C.

### 3.5. In Vitro Transfection Experiments

SKOV-3 or A549 cells were seeded on Costar 6-well plates (Corning Inc., Corning, NY, USA) at a density of 1.5 × 10^5^ cells per well and cultured in DMEM medium or RPMI-1640 as described before [24]. 30 min prior to transfection, the culture medium was replaced by 800 μL serum-free DMEM or RPMI-1640 in each well. Then Ch_2_-PEG_n_-CLs/pEGFP, CL/pEGFP, mPEG-CLs/pEGFP or F-Ch_2_-PEG_n_-CLs/pEGFP complexes (200 μL, containing 1 μg pDNA) was added to the wells respectively. Three wells were used for each lipoplex. After incubating for 5–6 h, cell culture medium was changed to DMEM or RPMI-1640 with serum and the cells were incubated for another 42–43 h. The transfected cells were observed under an inverted research microscope, Eclipse T*i* (Nikon Corporation, Tokyo, Japan). Then they were trypsinized with 0.25% trypsin-EDTA, centrifuged and resuspended with PBS. The cell suspensions were analyzed by a FACS Calibur flow cytometer (BD Biosciences, San Jose, CA, USA) to determine the transfection efficiency of the complexes.

### 3.6. Cytotoxicity of Ch_2_-PEG_n_ and Ch_2_-PEG_n_-CLs

Cytotoxicity of Ch_2_-PEG_n_ was evaluated in the SKOV-3 cell line by MTT assay as described previously [30]. Briefly, cells were seeded on 96-well plates (Corning Inc., Corning, NY, USA) in 100 μL medium at a density of 5 × 10^3^ cells per well. After overnight incubation, another 100 μL Ch_2_-PEG_n_ solutions at various concentrations (ranged from 1 to 200 μM) were added to the wells. The cells were incubated for another 24 h. Then 20 μL MTT solution (5 mg/mL in saline) was added to each well. After culturing at 37 °C for 4 h, the medium was removed. 160 μL DMSO was added to each well to dissolve formazan crystals. The absorbance was measured at 570 nm on a Multiskan MK3 microplate reader (Thermo Fisher Scientific Inc., Waltham, MA, USA). Untreated cells were used as controls. The relative cell viability compared with control was calculated based on the following equation: Relative cell viability (%) = (*A_treated_*/*A_control_*) × 100.

### 3.7. Statistical Analysis

Statistical analysis was performed using Student’s Independent-Samples *t*-Test on SPSS (V 19.0, IBM Corp., Armonk, NY, USA). All the statistical tests were two-sided. *p* < 0.05 was considered as statistical significant difference.

## 4. Conclusions

In this manuscript, a series of Ch_2_-PEG_n_ at different PEG molecular weights (600, 1000 and 2000) were successfully synthesized. Ch_2_-PEG_n_-CLs containing various Ch_2_-PEG_n_ presented different particle size, zeta potential and *in vivo* transfection efficacy, and Ch_2_-PEG_600_-CLs exhibited the strongest GFP expression in SKOV3 cells due to its highest zeta potential. However, Ch_2_-PEG_600_-CLs also had the highest *in vitro* cytotoxicity. After introduction of a folate ligand, the targeting efficacies and optimized ligand densities of F-Ch_2_-PEG_n_-CLs still depended on the molecular weights of PEG. In sum, Ch_2_-PEG_n_-CLs are promising carriers for gene delivery. The current work demonstrates the possibility of utilizing Ch_2_-PEG_n_ for gene delivery, and a corresponding systematic investigation of this study would benefit the future development of Ch_2_-PEG_n_-CLs-based gene delivery systems.
